# Patient Adherence to a Mobile Phone–Based Heart Failure Telemonitoring Program: A Longitudinal Mixed-Methods Study

**DOI:** 10.2196/13259

**Published:** 2019-02-26

**Authors:** Patrick Ware, Mala Dorai, Heather J Ross, Joseph A Cafazzo, Audrey Laporte, Chris Boodoo, Emily Seto

**Affiliations:** 1 Institute of Health Policy, Management and Evaluation Dalla Lana School of Public Health Toronto Toronto, ON Canada; 2 Centre for Global eHealth Innovation Techna Institute University Health Network Toronto, ON Canada; 3 Ted Rogers Centre for Heart Research University Health Network Toronto, ON Canada; 4 Department of Medicine University of Toronto Toronto, ON Canada; 5 Peter Munk Cardiac Centre University Health Network Toronto, ON Canada; 6 Institute of Biomaterials and Biomedical Engineering University of Toronto Toronto, ON Canada; 7 Canadian Centre for Health Economics Toronto, ON Canada

**Keywords:** telemonitoring, mHealth, adherence, heart failure

## Abstract

**Background:**

Telemonitoring (TM) can improve heart failure (HF) outcomes by facilitating patient self-care and clinical decision support. However, these outcomes are only possible if patients consistently adhere to taking prescribed home readings.

**Objective:**

The objectives of this study were to (1) quantify the degree to which patients adhered to taking prescribed home readings in the context of a mobile phone–based TM program and (2) explain longitudinal adherence rates based on the duration of program enrollment, patient characteristics, and patient perceptions of the TM program.

**Methods:**

A mixed-methods explanatory sequential design was used to meet the 2 research objectives, and all explanatory methods were guided by the unified theory of acceptance and use of technology 2 (UTAUT2). Overall adherence rates were calculated as the proportion of days patients took weight, blood pressure, heart rate, and symptom readings over the total number of days they were enrolled in the program up to 1 year. Monthly adherence rates were also calculated as the proportion of days patients took the same 4 readings over each 30-day period following program enrollment. Next, simple and multivariate regressions were performed to determine the influence of time, age, sex, and disease severity on adherence rates. Additional explanatory methods included questionnaires at 6 and 12 months probing patients on the perceived benefits and ease of use of the TM program, an analysis of reasons for patients leaving the program, and semistructured interviews conducted with a purposeful sampling of patients (n=24) with a range of adherence rates and demographics.

**Results:**

Overall average adherence was 73.6% (SD 25.0) with average adherence rates declining over time at a rate of 1.4% per month (*P*<.001). The multivariate regressions found no significant effect of sex and disease severity on adherence rates. When grouping patients’ ages by decade, age was a significant predictor (*P*=.04) whereby older patients had higher adherence rates over time. Adherence rates were further explained by patients’ perceptions with regard to the themes of (1) performance expectancy (improvements in HF management and peace of mind), (2) effort expectancy (ease of use and technical issues), (3) facilitating conditions (availability of technical support and automated adherence calls), (4) social influence (support from family, friends, and trusted clinicians), and (5) habit (degree to which taking readings became automatic).

**Conclusions:**

The decline in adherence rates over time is consistent with findings from other studies. However, this study also found adherence to be the highest and most consistent over time in older age groups and progressively lower over time for younger age groups. These findings can inform the design and implementation of TM interventions that maximize patient adherence, which will enable a more accurate evaluation of impact and optimization of resources.

**International Registered Report Identifier (IRRID):**

RR2-10.2196/resprot.9911

## Introduction

### Background

Heart failure (HF) telemonitoring (TM) interventions are designed to transform traditional HF management from one of episodic care (during periods of symptom exacerbation or scheduled follow-up visits) to one of continuous management, extending into patients’ daily lives. TM systems enable patients to take home readings (eg, weight, blood pressure, pulse rate, oxygen saturation, and symptoms) [1] which then get transmitted to clinicians at a remote location [2]. The main outputs of this data transfer are threefold. First, the act of taking regular measurements instills in patients a sense of active participation in their care while providing information required to engage in self-care [3,4]. Second, timely data transmission enables clinicians to catch symptom exacerbations early and allow for remote intervention [5]. Finally, even in the periods of patient stability, longitudinal data collected by TM systems provide a more holistic picture of patients’ condition which can improve the quality of clinical decisions [6]. According to several meta-analyses, these mechanisms work together to improve quality of life and reduce mortality and health care utilization compared with the standard of care without TM [2,7-10]. However, large and well-designed randomized controlled trials (RCTs) have reported null or mixed results which cannot be ignored [11-14]. We have previously made the case that inconsistencies in the evidence can be explained, in part, by varying fidelity with which interventions are implemented in trials, including the degree to which patients adhere to taking prescribed home readings [15].

Despite the importance of ensuring consistent patient adherence over the course of a TM intervention, there is a dearth in the literature on this topic [16,17] and existing knowledge is difficult to generalize. First, although systematic reviews describe general trends of adherence as starting high in the early months and dropping off over time, there is significant heterogeneity with overall rates between 40% and 90% being reported across studies [3,16]. Second, much of the remote monitoring literature on adherence relates to interactive voice response (IVR)–based interventions with much fewer studies related to newer forms of TM that leverage devices already familiar to patients (eg, mobile phones) [3]. Third, adherence is defined and measured inconsistently across studies with many simply reporting engagement with the technology (eg, taking a single measure) which does not always encompass the full set of patient behaviors needed to optimize the intervention’s mechanisms of action [18,19]. Finally, the phenomenon of patient adherence is typically measured in the context of RCTs, limiting the understanding of patient adherence within real-world TM contexts which may be less likely to limit patient use of an intervention to a predefined study period.

### Study Objectives

In August 2016, an HF TM program was deployed as part of the standard of care in a specialty heart function clinic in Toronto, Canada. A previously published case study of this program’s implementation described and explained clinician adoption as well as the degree of integration within the clinic [20]. This study aimed to describe the patient perspective within the case study. The objectives were to (1) quantify the degree to which patients adhered to taking prescribed home readings and (2) explain longitudinal adherence rates based on the duration of program enrollment, patient characteristics, and patient perceptions of the TM program.

## Methods

### Study Design

The study used a mixed-methods explanatory sequential design whereby overall and monthly patient adherence rates were first analyzed over a 1-year period. These adherence rates subsequently informed the sampling strategy for semistructured interviews with the objective of explaining overall adherence rates. Additional explanatory data were collected including questionnaires and reasons for leaving the program, all of which were triangulated with the interview findings to explain adherence rates.

The methods used to explain adherence were guided by the unified theory of acceptance and use of technology 2 (UTAUT2) which outlines how 7 constructs influence consumers’ intention to use a technology [21]. These constructs, whose definitions have been adapted to facilitate their operationalization within this study, include the following: (1) *performance expectancy* (the degree to which using the TM system is perceived to provide benefits for patients and is analogous to *relative advantage* in the diffusion of innovation literature and *perceived usefulness* in the technology acceptance model), (2) *effort expectancy* (the degree of ease associated with patients using the TM system), (3) *facilitating conditions* (patients’ perception that there are resources available to support their use of the TM system), (4) *social influence* (the extent to which others important to the patients [eg, family and friends] support the use of the TM system), (5) *hedonic motivation* (the fun or pleasure derived from using the TM system), (6) *price value* (patients’ cognitive trade-off between the benefit of using the TM system and the monetary and time costs to them), and (7) *habit* (the extent to which patients take their required readings automatically because of learning). The UTAUT2 also proposes that the influence of these 7 constructs on behavioral intention to use technology is modified by age, gender, and experience with using the technology [21].

Key methods for this study have been published in a protocol for a larger quality improvement program evaluation [22] which has been approved by the University Health Network (UHN) Research Ethics Board (16-5789). This approval included the analysis of all data collected as part of the standard of care (ie, TM usage data). Informed consent was obtained by patients who completed questionnaires and interviews.

### The Intervention

#### Telemonitoring Technology

**Figure 1:**
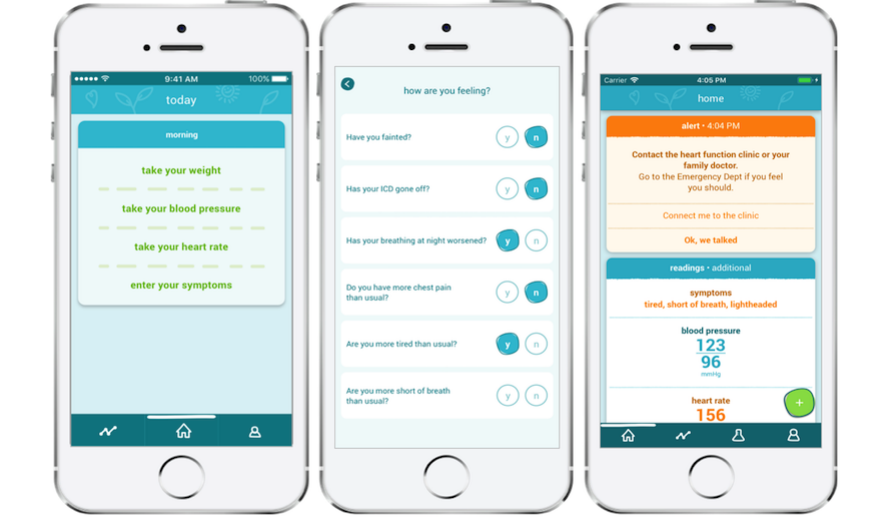
Screens of the Medly app showing the incomplete morning card with required readings, the symptoms questionnaire, and personalized self-care feedback after all 4 readings were taken and processed by the algorithm.

The central component of the *Medly* TM program is the *Medly* smartphone app that patients use to take weight, blood pressure, and heart rate readings as well as record their symptoms using a “yes/no” questionnaire composed of 5 to 11 “yes/no” questions. Patients are instructed to take these 4 readings daily within 30 min of each other before 12 pm. If this is done, the recorded data get processed by a clinically validated algorithm embedded in the app [23], which has been contextualized according to each patient’s target thresholds for each of the 4 readings. If the algorithm identifies that key readings are within the acceptable range, the reading values are presented on the screen with a message telling patients that their readings are fine on that day. However, if the algorithm identifies that key readings are out of range or that there is a worrying trend in weight gain, the app generates and displays self-care feedback messages which are highlighted in a different color depending on the determined urgency (Figure 1). Types of self-care feedback messages include the following: informing patients when they are outside their normal range, instructing them to take their prescribed diuretic medication, and suggesting when to contact their care providers or go to the emergency department. Similarly, clinicians also receive alerts when readings are out of range which they can receive via email or view within the clinician-facing *Medly* dashboard.

Other features of the *Medly* app include the ability to view graphical trends of each reading’s values and to assist with adherence, an automated phone call to their primary phone line (personal mobile phone or home landline) to remind patients if they have not yet taken morning readings by 10 am. This feature can be disabled at the patient’s request. The development of *Medly* features aimed at promoting patient self-care was guided by the Connelly Framework for Self-Care in Chronic Illness [24] (a derivation of the health belief model [25]) using an iterative user-centered design process which included a formal needs assessment [26] and multiple rounds of usability testing. Further description of the *Medly* program can be found elsewhere [20,22,27].

#### Program Enrollment and Onboarding

As the *Medly* program is offered as part of the standard of care, enrollment is decided jointly between patients and their treating cardiologist during a follow-up appointment or after an inpatient hospital stay. After the treating cardiologist explains the *Medly* program and the patient agrees to participate, they are immediately escorted to a private room where they receive training on how to use the technology. A part of this training includes highlighting the importance of taking daily readings.

#### Ongoing Monitoring

Throughout their participation in the *Medly* program, patients are expected to complete the 4 daily morning readings and to follow the self-care feedback displayed in the app. If clinical alerts are triggered, a designated clinician at the heart function clinic reviews these alerts as soon as possible. Most clinical alerts result in a call being made to the patient to obtain more information or to provide relevant clinical guidance.

#### Offboarding

Offboarding refers to the process of ending a patient’s participation in the *Medly* program. Unlike many TM interventions, the *Medly* program does not have a predefined end date. Thus, as with most medical interventions, patients remain enrolled for as long as there is a perceived clinical benefit. Patients or clinicians can, at any time, initiate a conversation about the appropriateness of the *Medly* program as part of a patient’s treatment plan. Once the joint decision to offboard a patient is made, patients return any equipment they borrowed (mobile phone or peripheral devices) which get recycled and used for future participants.

#### Adaptations to the Program Since Its Launch

When the program first launched in August 2016, patients were provided with a *Medly* kit which included a smartphone with a data plan and with the *Medly* app already downloaded along with a Bluetooth-enabled weight scale and blood pressure cuff. This enabled data from the peripheral devices to be transmitted directly and automatically to the *Medly* app. Training and ongoing technical support was provided by an analyst from the hospital’s telehealth department, and the triage of clinical alerts was done by nurse practitioners on staff at the clinic. Since program launch, 2 key changes have been implemented to enable the sustainability and scalability of the program. First, in January 2018, it became possible for patients with iPhones to download the *Medly* app on their own smartphones and to use their personal weight scales and blood pressure cuffs (the app is now also available for Android users; however, this option was not available at the time of data analysis). Patients without Bluetooth-enabled peripheral devices manually entered readings directly into the app. Second, as of May 2018, a *Medly* coordinator role was created whereby a registered nurse took over the role of triaging clinical alerts in addition to providing frontline technical support. Details and rational for these changes have been published elsewhere [27].

### Measuring Patient Adherence

As patients are instructed to take weight, blood pressure, heart rate, and symptom readings every morning (these 4 readings are required for the *Medly* algorithm to generate self-care instructions for patients and alerts for clinicians), adherence was defined as the proportion of days patients took all 4 morning readings over the total number of days they were enrolled up to 1 year. Owing to ongoing enrollment, not all patients had completed 1 year at the time of analysis. Thus, varying durations were accounted for in the proportion denominator. Similarly, monthly adherence rates were calculated as the number of completed morning readings over each 30-day period following the date of their enrollment up to 1 year. Proportions were multiplied by 100 to get monthly adherence rates expressed as a percentage of prescribed completed readings. To further understand patient engagement, we also calculated incomplete adherence rates which were defined as the percentage of days patients took at least 1 reading but not all 4 (such that, although data are transmitted, no clinical alerts or patient feedback is generated). Usage data required to determine adherence rate were collected between August 2016 and October 2018 and extracted from the *Medly* program server.

### Explaining Patient Adherence

#### Quantitative Data and Analyses

##### Explanatory Variables

Patient demographic variables were collected to characterize the patient population using a questionnaire administered immediately after program enrollment to patients who provided informed consent (n=174). Simple linear regression for full and incomplete adherence over time was performed. In addition, because adherence was collected using repeated measures over a 12-month period, a panel multivariate regression approach was used to determine the impact of time on adherence when controlling for key variables. Preliminary diagnostics included the Hausman and Lagrange multiplier to choose between pooled ordinary least squares, fixed effects, or random effects models and the Breusch-Pagan test to detect the presence of heteroscedasticity [28]. Ultimately, random effects models with cluster-robust standard errors (to adjust for the presence of heteroscedasticity) [29] proved best suited for the dataset. Selected explanatory variables for the multivariate regression included age categorized by decade and sex (both moderating variables in the UTAUT2) [21], and New York Heart Association functional classification (NYHA class), a subjective measure of HF symptom severity based on the hypothesis that sicker patients may benefit more. Data for baseline NYHA class (sometimes documented as a range), age, and sex were extracted from patients’ chart in the hospital electronic medical record.

##### Patient Questionnaire

As part of a larger questionnaire used in an impact evaluation [22], consenting patients responded to questions about their satisfaction with the *Medly* program at 6 and 12 months, offering an opportunity to triangulate these quantitative findings with results from patient interviews (described below). Items in the satisfaction questionnaire could be classified according to the key UTAUT2 constructs of *performance expectancy* (3 items) and *effort expectancy* (4 items); no questionnaire items could be classified within the remaining UTAUT2 constructs and thus were not quantitatively assessed.

Descriptive statistics for the questionnaire responses, adherence rates, and linear regressions were performed using SPSS version 24 (IBM Corporation). Multivariate regression analyses were conducted in RStudio v.1.0.153 (RStudio Inc) using the “plm” package [30]. For all statistical tests, a *P* value of less than .05 was used to indicate statistical significance. Temporal trends were graphically represented using Microsoft Excel (Microsoft Corporation).

#### Qualitative Data and Analyses

##### Reasons for Offboarding

Reasons leading to patients being offboarded were recorded in the *Medly* coordinator’s records as part of the standard offboarding procedures. These reasons were qualitatively analyzed and classified into themes before being transformed into a count for each category.

##### Patient Interviews

Semistructured interview guides, which were developed to understand the patients’ experience in the *Medly* program, included probes based on the constructs in the UTAUT2. Participants were identified using a purposeful sampling approach to ensure a variety of opinions and to reach information saturation [31]. Variables considered in this sampling approach were age, sex (age and gender are both moderators in the UTAUT2), overall adherence rates, and time since enrollment. The latter involved selecting patients who had been enrolled for different durations, including baseline (to understand initial perceptions without actual experience), approximately 1 month (the intervention was still fresh but patients had been using it long enough to experience benefits and barriers to use), and approximately 6 and 12 months (to align with questionnaire administration and assess patients’ perceptions after longer term use). Interviews were recorded and took place in a private room during a scheduled clinic visit or over the telephone.

Interview transcripts were analyzed by 2 independent investigators (PW and MD) using the framework method [32]. This approach involved a first round of largely deductive thematic analysis using an initial coding framework based on the UTAUT2 constructs. PW and MD met to discuss results of the first round and to agree upon subthemes within those constructs. Next, a second round of independent coding was done using the updated coding framework, which was followed by a meeting to discuss contradictory codes and passages. The management of source documents and coding was accomplished with the help of NVivo version 11 (QSR International).

## Results

### Characteristics of Study Participants

Participants of the *Medly* program were predominantly male (184/232, 79.3%) and had an average age of 57.6 (SD 16.0) years. Other demographics presented in Table 1 are representative of the patient characteristics typically followed in this urban heart function clinic. With regard to HF severity, approximately half experienced relatively mild HF symptoms daily with 48.5% (109/225) having an NYHA class of 2 or less at the time of program enrollment and the average left ventricular ejection fraction of patients was 32.1 (SD 13.2). Most patients included in this analysis (201/231, 87%) used the full kit version of the *Medly* system, 8.2% (19/231) used their personal smartphone but were given peripheral devices by the clinic, and the remaining 4.8% (11/231) used their personal smartphone and either purchased or used their own weight scales and blood pressure cuffs. The option for patients to use their own equipment started approximately 1.5 years after the launch of the program [27].

### Overall and Longitudinal Adherence Rates

**Figure 2:**
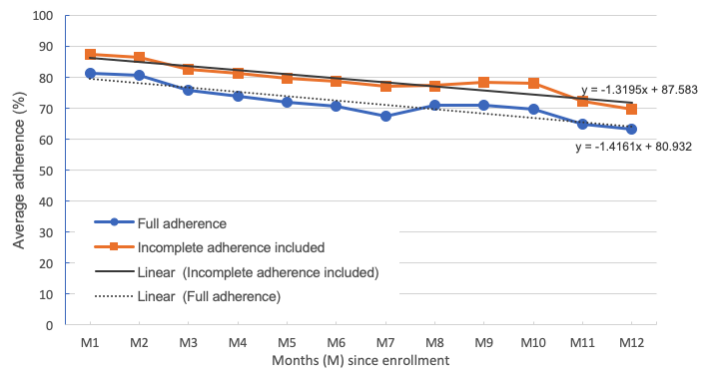
Average full adherence rates compared with adherence rates which include incomplete adherence over time.

The average overall adherence rate for the 231 patients included in the analysis was 73.6% (SD 25.0), indicating that the average patient completed their prescribed morning readings 5 days per week over the course of their enrollment in the program. When considering days where patients took at least 1 but fewer than all 4 morning readings (ie, including incomplete adherence), the average rate was 80.0% (SD 21.7). Longitudinal examination of monthly adherence rates shows a relatively high average adherence in the first month of 81.2% (SD 23.0) with a gradual decline to 63.1% (SD 37.0) after 12 months of enrollment (see Figure 2). Outputs of the simple linear regression indicates that time is a significant predictor of adherence (beta=−1.42, *P*<.001) with each month since enrollment accounting for a 1.4% decrease in adherence.

**Table 1:** Characteristics of patients included in the quantitative analysis of overall and longitudinal adherence.

Characteristic		Statistics
Age (years), mean (SD)		57.6 (16.0)
**Age (years; categorical), n (%)**		
	70 or more	60 (25.9)
	60-69	56 (24.1)
	50-59	50 (21.6)
	40-49	34 (14.7)
	39 or less	32 (13.8)
**Sex, n (%)**		
	Male	184 (79.3)
	Female	48 (20.7)
**Ethnicity, n (%)**		
	White	115 (66.0)
	Black	14 (8.0)
	Asian	21 (12.1)
	Other	24 (13.8)
**Rurality, n (%)**		
	Urban	100 (58.1)
	Suburban	49 (28.5)
	Rural	23 (13.4)
**Place of birth, n (%)**		
	Canada	85 (48.9)
	Elsewhere	89 (51.1)
**Highest education achieved, n (%)**		
	Less than high school	13 (7.5)
	High school	34 (19.5)
	College or university	127 (73.0)
**Income in Can $, n (%)**		
	<$15,000	26 (15.1)
	$15,000-$49,999	57 (33.1)
	>$50,000	58 (33.7)
	Preferred not to answer	31 (18.0)
**Work, n (%)**		
	Working full time	35 (20.2)
	Working part time	17 (9.8)
	Retired	87 (50.3)
	Unemployed/homemaker	14 (8.1)
	Other	20 (11.6)
**Supplementary health insurance, n (%)**		
	Yes	104 (60.8)
	No	67 (39.2)




**New York Heart Association functional classification,** **n (%)**		
	2 or less	109 (48.5)
	2-3	48 (21.3)
	3 or more	68 (30.3)
Left ventricular ejection fraction, mean (SD)		32.1 (13.2)
**Have a smartphone, n (%)**		
	Yes	119 (70.4)
	No	50 (29.6)
**Comfort with smartphone, n (%)**		
	Not comfortable	5 (4.0)
	Somewhat comfortable	24 (19.2)
	Comfortable	47 (37.6)
	Very comfortable	49 (39.2)
**Equipment used by patients, n (%)**		
	Full *Medly* kit	201 (87.0)
	Patients used personal phone and were provided with peripherals	19 (8.2)
	Patients used all personal equipment	11 (4.8)

### Quantitative Results Explaining Adherence

#### Multivariate Regression

**Figure 3:**
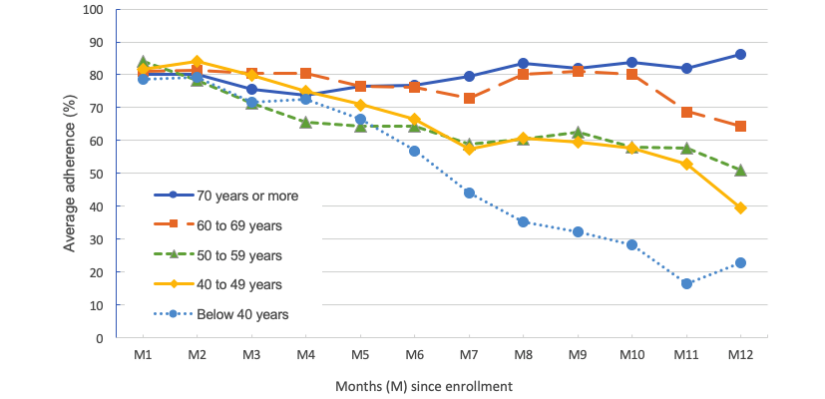
Average adherence rates over time by age group showing higher adherence over time for older age groups.

Random effects multivariate regression with cluster-robust standard errors was performed as described in the Methods section. The results, presented in Table 2, confirm a significant effect of time on adherence with each passing month starting after the second month of program enrollment. Patient age was a significant predictor of adherence (*P*=.04); the positive coefficient indicates that adherence rates were higher with each increasing age category such that older patients maintained higher adherence over time. Figure 3 shows adherence rates over time with regard to the age groups included in the regression model. Disease severity (NYHA class) and sex were not significant predictors of adherence.

**Table 2:** Random effects multivariate regression with cluster-robust standard errors (SE) showing the effect of time, sex, New York Heart Association (NYHA) class, and age on average adherence.

Variables	Coefficient (beta)	SE	*P* value
Intercept	87.57	4.03	<.001
Month 1	Ref^a^	—^b^	—
Month 2	-1.27	1.98	.52
Month 3	-5.63	2.36	.02
Month 4	-8.21	2.80	.004
Month 5	-9.84	2.85	<.001
Month 6	-12.65	3.05	<.001
Month 7	-15.87	3.33	<.001
Month 8	-12.45	3.32	<.001
Month 9	-13.71	3.63	<.001
Month 10	-15.11	4.20	<.001
Month 11	-19.55	4.84	<.001
Month 12	-20.98	5.13	<.001
Sex	-2.33	5.61	.68
NYHA class	-0.34	2.60	.90
Age	3.49	1.68	.04

^a^Month 1 is the reference category to which all other levels of the time variable (months 2 to 12) are compared in the multivariate regression model.

^b^Not applicable.

#### Patient Questionnaire

Results from the patient questionnaires show that a clear majority of patients perceived value in using the *Medly* system after 6 months with 90.6% (87/96) agreeing or strongly agreeing with the statement that the TM system is important for managing their HF and 87.4% (83/95) agreeing with the statement that it would be useful for them to continue using the system (Table 3). The percentage of patients who agree with these same statements increased to 95.8% (46/48) and 93.9% (46/49) after 12 months, respectively. Responses related to *effort expectancy* at 6 months similarly show a high level of agreement with 92.7% (89/96) of patients agreeing with the statements that the TM system was easy to use and to learn how to use it. Perceptions of ease of use remained consistent with 89.4% (42/47) and 91.8% (45/49) agreeing with these same statements at 12 months.

**Table 3:** Patient perceptions of the benefits and effort of using the *Medly* TM system at 6 and 12 months postenrollment.

Item in questionnaire		Agree or Strongly agree, n (%)	
		6 months	12 months
**Performance expectancy**			
	The monitoring system is important for managing my heart failure	87 (90.6)	46 (95.8)
	I think using the monitoring system improved my health	65 (70.7)	36 (75.0)
	It would be useful for me to keep using the monitoring system	83 (87.4)	46 (93.9)
**Effort expectancy**			
	Learning to operate the monitoring system was easy for me	89 (92.7)	45 (91.8)
	I found the monitoring system to be easy to use	85 (92.4)	42 (89.4)
	Taking my blood pressure at home was easy	93 (96.9)	47 (95.9)
	Taking my weight was easy	93 (96.9)	47 (95.9)

**Table 4:** Classification of reasons for patient offboarding.

Reason for offboarding		Statistics, n (%)
**Clinician–initiated offboarding**		
	Received heart transplant or surgical repair of the heart	14 (22.9)
	Switched to more invasive form of remote monitoring (eg, CardioMEMS)	5 (8.1)
	Patient recovered ventricular function	4 (6.5)
	Significant change in health status (eg, shift to palliative care)	6 (9.8)
	Patient was not compliant with taking readings or with following clinician instructions	3 (4.9)
**Patient–initiated offboarding**		
	Not interested in participating or a belief that the benefits are not worth the effort	5 (8.1)
	Stress caused by taking daily readings	4 (6.5)
	Life circumstances (eg, shift work and sick relatives)	2 (3.2)
	Poor eyesight	1 (1.6)
	Other (eg, unknown, moved provinces)	5 (8.1)
Mortality		12 (19.6)

### Qualitative Results Explaining Adherence

#### Reasons for Offboarding

Of the 61 patients who left the *Medly* program during the study period, 52% (32/61) were offboarded because a change in their HF condition made it such that the *Medly* program would no longer be a beneficial part of their care plan. A total of 3 patients were offboarded because they were not adhering to taking measures or following clinician instructions (Table 4). A further 28% (17/61) of patients chose to leave the program because of a lack of interest or a feeling that the benefits of enrollment were not worth the effort, that daily monitoring was causing stress, and for other unknown reasons. Finally, 20% (12/61) of the offboarding were because of patient death. These deaths were attributed to the severity and natural progression of HF.

#### Interview Findings

##### Interview Participant Characteristics

The interviewed participants (n=24) largely matched the distribution of age and sex of the larger patient sample as shown in Table 5. The patients interviewed had overall adherence rates ranging between 22.2% and 98.6% and were interviewed at various times since program enrollment. This included 17% of patients (4/24) being interviewed the day they were onboarded and 2 patients who agreed to participate after deciding they wanted to leave the program.

**Table 5:** Participant characteristics for semistructured interviews.

Participant ID	Sex	Age at enrollment (years)	Time of interview since enrollment (month)	Average adherence rate (%)
HFpro009	M	76	12	92.2
HFpro011	M	72	12	80.0
HFpro018	M	60	6^a^	90.0
HFpro019	M	46	3	30.3
HFpro027	M	59	6	82.3
HFpro028	M	67	6	93.1
HFpro037	F	62	0	90.3
HFpro038	M	63	6	97.5
HFpro048	M	44	1^a^	96.7
HFpro052	M	83	6	70.3
HFpro059	M	76	6	54.2
HFpro060	F	81	6	96.3
HFpro061	M	62	6	45.0
HFpro064	M	45	6	67.9
HFpro089	M	57	9	72.2
HFpro091	F	61	12	94.4
HFpro107	M	54	6	62.8
HFpro109	M	41	1	68.3
HFpro129	M	22	1	22.2
HFpro131	F	71	0	87.0
HFpro154	M	50	1	96.1
HFpro157	F	45	0	98.6
HFpro158	F	52	0	82.4
HFpro168	F	65	6	90.6

^a^Interview conducted after offboarding.

##### Interview Themes

Interview themes were classified according to UTAUT2 constructs of *performance expectancy,* *effort expectancy*, *facilitating conditions*,*social influence*, and *habit*; no statements related to *hedonic motivation* or *price value* were identified by the coders. No overarching patterns emerged in the themes based on patients’ age, sex, or time since enrollment. Therefore, the themes and representative quotes discussed in the following sections predominantly help distinguish between high and low adherers.

###### Performance Expectancy

This theme refers to the perceived benefits, both expected and experienced, of being part of the *Medly* program. Subthemes included (1) self-management support, (2) peace of mind, (3) relationship with care team, and (4) lack of context.

####### Self-Management Support

The most commonly mentioned benefit of the *Medly* program is that it supports patients in their ability to self-manage their HF. Participants discuss how the system, by enabling them to take daily readings, keeps them accountable and provides guidance in their self-care tasks:

I rarely ever took my weight which was a big issue getting admitted into the hospital because I retain so much water. So yeah, it’s been helpful for monitoring things that I normally wouldn't…It keeps me on track and lets me know if I need to take medication that I normally don’t have to take it...I had the expectation that it would be helpful for me because it keeps me on a routine and it's lived up to those expectations.

HFpro154

Underpinning this self-management support is the immediateness of the patient self-care feedback which allows patients to plan for their day around the results of readings they have just taken:

I can start my day off with knowing that I’ve got to be extra careful…I’m going to plan my day from what Medley is telling me. That’s how it helps me every morning, I know what to do and what not to do for the rest of the day.

HFpro089

####### Peace of Mind

The automated self-care feedback works alongside clinician monitoring to provide many patients with peace of mind. For some, this peace of mind brings a heightened sense of confidence when they are trying to decide if their symptoms are bad enough to warrant a trip to the hospital.

I’m very diligent...I basically rely on it. I just love the peace of mind that it represents. When you’re as sick as I was, it’s good to have a big brother or big sister out there.

HFpro107

It gives me comfort [knowing that] somebody’s watching over me, I don’t have to go to the hospital all the time.

HFpro052

####### Relationship With Care Team

Patients who had been in the program for longer periods said that it improved their relationship with clinical members of their care team:

At first it didn’t bother me [that I didn’t have a lot of interaction] but now actually you gain trust, you know, a relationship with the person on the other end...When [the Medly nurse] calls sometimes, we’re talking for 20 minutes and she’s really getting a full history of things because you just can’t get a full history on a minute conversation.

HFpro089

However, for some, this closer relationship helps explain lower adherence in patients who did not like the idea of clinicians being able to see transgressions of daily life:

There is a feeling, and it sort of upsets me or disturbs me that they know [everything I do]. If I want to go out on a binge and watch a soccer game or a hockey game and eat lots …they’re going to know because my weights going to go up and so there’s a fear of, “Oh God, I’m going to be told off.” Will it stop me [from] doing that? No, what will stop me doing it is the fact that it’s bad for my health… I forget to take my weight [laughs] because there’s a feeling that they’re looking.

HFpro061

####### Lack of Context

Patients with lower overall adherence rates expressed the opinion that the readings, particularly the symptoms, do not accurately capture the full context of their health status. Consequently, some felt that the self-care feedback messages did not always reflect how they were feeling and they eventually learned that they should not immediately act on some alerts:

[The feedback messages] are a little bit too alarming... because sometimes they say “have somebody take you immediately to emergency,” and usually it turns out it's okay, so I got used to that.

HFpro009

###### Effort Expectancy

Patient uptake and adherence can also be explained by the perceived efforts involved in using the system. Subthemes included (1) usability and (2) technical difficulties.

####### Usability

The qualitative findings mirror those from the questionnaire insofar as most patients found the *Medly* system easy to learn and use. Furthermore, some patients were frequent travelers and described how the portability of the system allowed them to continue taking their readings wherever they were:

I’m of the age where I’m not as computer literate with cellphones... But it was fine, it’s easy. If I can learn it, it’s pretty easy to learn.

HFpro027

It’s been all over Canada with me… we just throw it in the car. I even took my weight at Tim Horton’s first thing in the morning and that was in Edmonton. We just left the hotel and it was in the car and…before we left [on] the road I said I didn’t take my weight. And my wife went out and got it, hooked it up to the Wi-Fi at the Tim Hortons and bam, [I] took it right there.

HFpro089

A minority of patients described some difficulty using the peripheral devices and the cognitive effort in trying to decide how to accurately answer the “yes/no” symptom questions:

The equipment is highly sensitive. For me I have dizziness constantly because of my medications and my low blood pressure and my heart condition. So, if I sway or move on the scale, the scale has different readings…The scale is very narrow... and I’m a very wide guy and I need to have my feet spread apart in order to be stable on the scale... The same with my arm, if I move my arm a little bit or anything, (it) will make the blood pressure monitor go into error mode and it's frustrating

HFpro064

With the [symptoms] questionnaire sometimes I’m sort of on the edge because it says “Are they worse?” Well no, they’re not any worse but sometimes I am like a little bit short of breath.

HFpro091

####### Technical Issues

The quality of the system was perceived as high across all patients and time points. However, several patients, from various adherence levels, recounted experiencing technical issues, particularly related to Bluetooth connectivity between the peripheral devices and the smartphone.

There are times when I’m not impressed, because I weigh myself but it doesn’t record. And, then I get this call saying that I didn’t do it and it throws me off. But, generally, it’s okay. I’ve had, I think about three times where it’s misfired sort to speak...I think, “what did I do wrong?”

HFpro060

###### Facilitating Conditions

Facilitating conditions are the resources and support available to facilitate the use of *Medly*. Subthemes included (1) technical support, (2) automated adherence calls, and (3) informal caregivers.

####### Technical Support

The technical issues described did not seem to have severely impacted adherence rates because of the easy access to technical support services. In addition, the presence of comprehensive onboarding process helped with initial uptake:

I can’t see [a reason to not use Medly]. I mean, as far as running into technical difficulties, you’ve given me all the numbers, there’s people I can contact so I don’t foresee there being that big of a problem that I wouldn’t be able to work through it.

HFpro027

[The training was] a piece of cake...it was private, we were in a closed room, the information was face to face, the equipment was right there, it had hands-on... it was great.

HFpro018

####### Automated Adherence Calls

The automated call that is sent to patients if they have not taken their readings before 10 am, although sometimes described as annoying, was a facilitating condition expressed by many:

[Taking my readings] is what I do first thing in the morning before I get the phone call with the annoying ringing… I do [appreciate the call but]...I’m also a single dad of two kids so any opportunity that I possibly can to rest and sleep I take it... A text would be better than a phone call.

HFpro064

####### Informal Caregivers

Although the technology is designed to support the HF patient experience, some patients receive support from family members who help with all aspects of HF management, including reminding them to take their *Medly* readings:

I have a built-in monitor at home [laugh]...which is very, very beneficial because if [my wife] wasn’t there at all, you know, I’d probably be worse than I am, as far as habits are concerned. So, having that extra person, she polices me pretty good.

HFpro028

###### Social Influence

Social influence is the degree to which individuals in patients’ lives were supportive of the use of TM. Patient responses revealed that their family and friends overwhelmingly support their participation in the *Medly* program:

Everybody knows [I use Medly]. I write about it all the time...everybody is very envious that I’m on this type of program and envious that I have doctors that care about me this much.

HFpro064

Some patients had family members who raised privacy concerns, but this does not seem to have impacted the willingness to participate:

Well, [my friends and family] think it’s great. There’s a few that think it’s kind of Big Brother [saying] “wow, they know a lot of information on you.” But their fear of Big Brother is kind of secondary to my doctor need[ing] to know what’s going on.

HFpro019

Finally, the fact that the *Medly* program was endorsed by a trusted clinician also appears to have been a motivating factor for patients:

[My cardiologist] is always very supportive saying, “[Medly]’s really doing a good job for you keeping you out of emergency.”

HFpro009

###### Habit

Evidence of the formation of a habit was more prominent in patients with higher adherence rates with many describing how taking measures eventually became a part of their delay routine. Once a habit was established, events or conditions breaking that routine explained why some readings were missed:

It’s part of a habit now. I don’t forget it. ...[It’s] automatic.

HFpro038

[When I forget] I think I can smell my wife making the coffee... The sense of smell is very strong …and it just beats the other senses out of my head that say “go weigh yourself first.” The smell of fresh coffee coming down the hallway, honestly that's the only time I would miss it.

HFpro089

Although the formation of a habit helps, it is not essential to ensure high adherence. One patient described the hassle of taking daily readings yet still maintained a high level of adherence (90.0%) throughout their enrollment. In this example, factors such as guilt and recognizing the importance of a behavior may be enough to motivate daily readings even if this behavior did not become automatic:

I don’t like doing it every day, it’s a bit of drag, because sometimes I want to sleep in and I feel kind of guilty because I haven’t got it done...I’m just sort of getting old and lazy and don’t want to do anything but that’s part of my regimen, I never miss.

HFpro018

## Discussion

### Principal Findings

This study has presented the findings of a mixed-methods study seeking to describe and explain patient adherence rates to taking daily prescribed home readings over a 1-year period in the context of a mobile phone–based HF TM program offered as part of the standard of care. Results found an average overall adherence rate of 73.6% and an average 1.4% drop in adherence with each passing month. The random effects model, which enabled repeated measures of monthly adherence (effect of time) to be included in the same regression as other demographic variables, found a significant effect of age on monthly adherence rates. Specifically, adherence rates were highest (and more consistent over time) for the older age group (70 years or more) and were progressively lower for each younger decade.

Additional methods employed could not fully explain the temporal decline in adherence, but they did provide evidence that patients’ perceptions of the program and other contextual factors contribute to explaining higher and lower adherence rates. Factors explaining patients’ motivation to adhere include the following: (1) perceived benefits of the program (self-management support, peace of mind, and improvement in clinical care), (2) ease of use, (3) a positive opinion of the program from family and friends, (4) supporting services (training and technical support), and (5) the ability to form a habit. Themes explaining low and imperfect adherence included the following: (1) technical issues, (2) life circumstances that interfered with a formed habit, and (3) a perception that the benefits of the program were suboptimal because of the system’s inability to adequately capture and communicate the full context of patients’ health state. These explanatory findings fit within the constructs of the UTAUT2 of *performance expectancy*, *effort expectancy*, *facilitation conditions*, *social influence*, and *habit*.

There were no findings related to the UTAUT2 constructs of *hedonic motivation* and *price value*; however, this is likely because of the context in which this study was conducted. First, although patients expressed numerous benefits, it remains that the use of TM systems occurs in the context of disease management and therefore is unlikely to be described as fun or enjoyable. Second, Canada has a public payer health system which means that patients did not have to pay out-of-pocket to use the technology. In addition, those who used their own smartphones and peripheral devices either already had that equipment or were assessed for their ability to pay or cover the costs through supplementation health insurance. Thus, patients were not put in a position of having to weigh the supplemental personal costs and benefits of being part of the *Medly* program.

Finally, although the principal aim of this study was to explain adherence using a definition based on the prescribed patient behavior needed to optimize program benefits, the finding that the incomplete adherence rate was 6.4% higher than full adherence should not be discounted. A certain percentage of these incomplete morning readings are likely due to the Bluetooth connectivity issues expressed by patients which would have prevented taking a weight or blood pressure reading until the issue could be fixed. Other possible explanations may include patients not recognizing or remembering the importance of taking the full set of readings. Alternatively, patients may make the decision to take measures that are most relevant based on how they are feeling (because of a high sense of self-efficacy for self-management) and may not necessarily lead to poorer outcomes. These hypotheses cannot be confirmed by the explanatory data generated in this study and should be empirically tested in future studies. The impact of adherence rates and health outcomes will be explored in a subgroup analysis of the upcoming impact evaluation of the *Medly* program [22].

### Comparison With Previous Work

#### Measuring Adherence

The findings from this study are in line with the literature review by Maeder et al, which found that adherence rates in home–based telehealth projects ranged from 40% to 90% and tended to be higher in earlier months before dropping off over time [16]. A recent and similar study looked at adherence to taking vital signs in TM interventions addressing various conditions and found an average adherence rate of 64.1% to scheduled daily readings. However, this study also found a trend toward increasing adherence after a steep initial drop off which is difficult to interpret alongside our results. The authors did not fully explain this initial drop off but hypothesized that patients may be encouraged to adhere only after longitudinal values could be generated and that they have had enough time to experience the value of the intervention [33]. In another study, adherence to completing IVR calls was 90% in HF patients, [34] but calls were only scheduled once per week, again, limiting comparison to a regimen that asks patients to take readings daily.

#### Explaining Adherence

The finding that older patients maintained a high level of adherence is seen in another remote monitoring study [34] but without explanation. The UTAUT2 proposes that the moderating impact of age is such that the effect of *effort expectancy* and *facilitating conditions* is strongest in older people [21]. In addition, it has been found that after a habit has been formed through repeated use, it becomes more difficult for changes in one’s external environment to override that behavior in older compared with younger people [21]. In other words, the ease of use of the *Medly* system and the availability of supporting services likely led to higher use in older patients which would have led to the formation of a habit. A habit which, once formed, would be more difficult to disrupt compared with younger patients. Although this may explain some of the differences between age groups, it is also possible that younger patients experience more potential distractors (work and dependent children) than older patients. Further research is required to understand the effect of age when it comes to adherence to TM interventions.

Factors explaining higher and lower adherence in this study were similar to the barriers and facilitators to TM, mobile health, and telehealth use described in the literature. With regard to *performance expectancy*, the literature cites similar perceived benefits including the degree to which the intervention improves health management (including both self- and clinician-directed management), peace of mind, and enhanced relationship between patients and clinicians [3,4,19,35-41].

Often-cited factors related to *effort expectancy* include user-friendliness of the equipment, technical barriers, health literacy or language barriers, and limited answer options [3,4,19,36-40,42]. The latter was seen in this study with patients who struggled with the “yes/no” format of symptoms.

In terms of *facilitating conditions,* studies support the availability of technical support services and features to help with remembering as important factors in technology use [3,19,37,38].

Similar factors related to *social influence* are discussed in a systematic review by O’Connor, which cites the lack of clinical endorsement as a barrier to patient uptake [40]. A survey study found that the construct of social influence contributed to explaining patients’ intention to use electronic health systems beyond what could already be explained by performance and effort expectancy [36]. In this study, we found overwhelming support for the use of *Medly* by family, friends, and the patient’s treating cardiologist. Thus, although social influence was likely not a strong enough factor in isolation, it probably contributed to higher adherence in the initial stages of enrollment.

Barriers such as failure of daily readings to become automatic and integrated into patients’ everyday tasks are cited in the literature [4,40] and were categorized in the construct of *habit* in our study. If an automatic habit is not created, the added energy of taking daily readings likely contributes to user fatigue over time.

This discussion is intended to highlight the prevalence of UTAUT2 themes in the literature. However, it is important to recognize that each TM intervention is different and may yield different experiences for patients. For example, a study by Fairbrother et al concluded that, although patients experienced peace of mind, the TM intervention did not increase the sense of ownership over their condition [43]. This contrasts with many of the patients in our study who described *Medly* as facilitating self-management. This is likely explained by the automated self-care feedback messages not part of the TM system in the Fairbrother study. This is an example of how different patient experiences can explain some of the heterogeneity of results across adherence studies. This study focused on patient perceptions because it is individuals’ experiences that are most likely to influence the degree to which they adhere to a TM program. The evaluation of the program’s outcomes, including quantitative measures for quality of life and self-care, is outside of the scope of this study and will be discussed in an upcoming publication [22].

### Limitations

Several limitations related to this study’s pragmatic design should be considered when interpreting the results. First, unlike TM interventions in RCTs, the *Medly* program was adapted over the 2-year period in which data collection occurred. As described elsewhere [27], these changes were made such that the essence of the intervention was maintained but it remains that some patients may have had different experiences.

Second, because enrollment in the intervention was not contingent on patients being part of a study, we were conscious of not overburdening patients with interviews at multiple time points. This decision meant that we did not collect qualitative data of individuals as they progressed through the program.

Third, reasons for offboarding were limited to the administrative data collected by clinicians and not all patients in the program had consented to being approached for an interview.

Fourth, the lack of strict inclusion criteria means that, by experimental standards, selection bias likely occurred such that enrolled patients were more likely to be engaged and not face language barriers. Furthermore, limiting the generalizability of findings are the demographics of the sample and the overrepresentation of male patients in the *Medly* program. This is consistent with other studies that find an under-representation of women enrolled in heart function clinics and clinical research despite a similar prevalence of HF among both sexes [44,45]. Although exploring the reasons for why fewer women have access to HF management interventions is outside the scope of this study, there is clearly a need for research into sex- or gender–based differences, including as it relates to uptake, use, and adherence to TM interventions. For instance, it is possible that the reason sex was not correlated with adherence rates in the regression analysis is because of a relatively small number of female participants in this study.

Fifth, the sample size of available data got progressively smaller with each passing month, and although this was accounted for in the regression analyses, it is likely that patients with strong negative opinions of the program left the program before the 12-month point (and thus did not complete the questionnaires).

Sixth, a previously published protocol [22] describes the use of quantitative methods for measuring patient adherence and interviews guided by the UTAUT2 to explain these adherence rates. Other data were collected as part of this pragmatic evaluation (ie, patient satisfaction questionnaire and reasons for offboarding) and were reported because they offer an opportunity to further explain patient adherence through triangulation with patient interviews. However, because the satisfaction questionnaire was not initially developed to explain adherence, it only contained items related to 2 of the 7 UTAUT2 constructs. Researchers conducting questionnaire–based work related to the UTAUT2 should consider using tools which include the validated items for that framework [21].

Finally, we did not have data allowing us to account for periods when patients were unable to take readings for legitimate reasons (eg, traveling, admitted in the hospital, and system down time), which would ideally be accounted for when measuring adherence. This limitation likely underestimates true adherence rates in the *Medly* program.

### Recommendations

On the basis of the findings from this study, we agree with recommendations from other studies that patients should receive comprehensive training and may benefit from refresher sessions aimed at reminding them of the proper use of the TM system, the benefits of the TM intervention, and the process for obtaining technical support when needed [6,16]. We also advocate for the involvement of supportive family members in those discussions and as part of the onboarding process. In addition, because many HF patients receive support from informal caregivers, further research into how best to incorporate that role within the design of TM systems would be beneficial.

Reminders (such as adherence calls) were found to be important in this study. Therefore, developers of TM systems should offer a range of options (eg, phone call, text, and app notification) that users can choose from based on their preferences such that these reminders do not become so disruptive that they opt to disable the feature.

Finally, study results offer important insights into how the user-centered design of TM systems should be conducted. Although there is value for scenario–based usability testing in laboratory environments, new TM systems should also be piloted in the real world with users of all age groups before full deployment. This is needed to allow TM designers to understand how patients use (or do not use) the system in the context of their existing habits and personal lives. In addition, although self-care messages can be simulated in a single usability testing session, it is preferable to give patients the opportunity to use a TM system over a period of time to evaluate the accuracy and appropriateness of self-care messages in response to fluctuations in their health state.

### Conclusions

This study has presented the results of a mixed-methods study aimed at explaining longitudinal adherence rates of patients enrolled in a mobile phone–based HF TM program. The study found that, on average, patients took weight, blood pressure, heart rate, and symptom readings for 73.6% of the days they were enrolled in the program. Results also showed a consistent decline in adherence over the 12 months which is further influenced by patients’ age such that patients in older age groups maintained higher and more consistent adherence rates throughout the study period whereas the declining rate of adherence became progressively more pronounced for younger age groups. Finally, interview findings indicated that the perceived benefits of the program, ease of use, social and technical support, and ability to form a habit around taking daily readings further explained levels of adherence. These findings can inform the design of TM interventions that maximize patient adherence. When implemented in the context of effectiveness trials, interventions with high fidelity of use will enable a more accurate evaluation of impact, and when implemented as part of the standard of care, they will ensure the optimization of resources and satisfaction among patient and clinician users.

## Abbreviations

HF: heart failure
IVR: interactive voice response
NYHA class: New York Heart Association functional classification
RCT: randomized controlled trial
TM: telemonitoring
UHN: University Health Network
UTAUT2: unified theory of acceptance and use of technology 2
